# Esophagogastric junction adenocarcinoma shares characteristics with gastric adenocarcinoma: Literature review and retrospective multicenter cohort study

**DOI:** 10.1002/ags3.12406

**Published:** 2020-10-26

**Authors:** Yu Imamura, Masayuki Watanabe, Eiji Oki, Masaru Morita, Hideo Baba

**Affiliations:** ^1^ Department of Gastroenterological Surgery Cancer Institute Hospital of Japanese Foundation of Cancer Research Tokyo Japan; ^2^ Department of Surgery and Science Graduate School of Medical Sciences Kyushu University Fukuoka Japan; ^3^ Department of Gastroenterological Surgery Kyushu Cancer Center National Hospital Organization Fukuoka Japan; ^4^ Department of Gastroenterological Surgery Graduate School of Medical Sciences Kumamoto University Kumamoto Japan

**Keywords:** esophageal adenocarcinoma, esophagogastric junction, gastric cancer, molecular subtype, prognosis

## Abstract

The incidence of esophagogastric junction (EGJ) adenocarcinoma has been gradually increasing in Asia, just like in Western countries a few decades ago. Despite recent advances in next‐generation sequencing and multimodal treatments, EGJ adenocarcinoma is still an aggressive malignancy with poor outcomes. Clinically, EGJ adenocarcinoma can be separated into Barrett's adenocarcinoma and cardiac adenocarcinoma, with frequent similarities observed. Barrett's adenocarcinoma is likely to be of gastric origin in terms of its premalignant background, risk factors, and stem cell regulators. Recent comprehensive genomic analyses suggest that immunotherapy may be essential for high‐level microsatellite instability (MSI‐H)‐ and Epstein‐Barr virus (EBV)‐associated subtypes, and against the immunosuppressive phenotype in genomically stable (GS) subtypes, in the treatment of EGJ and gastric adenocarcinoma. Although the chromosomal instability (CIN) subtype dominates EGJ adenocarcinoma, there is still a need to investigate the other molecular subtypes and their targets. Because of the distinctive characteristics of tumor location of EGJ adenocarcinoma, we also described the results of a multicenter cohort study of EGJ adenocarcinoma, comparing Siewert type I (distal esophagus), II (cardia of the stomach), and III (subcardia) tumors. We show that type I tumors were frequently accompanied by Barrett's esophagus (78%, *P* < .0001), with a significantly unfavorable outcome (multivariate EGJ‐cancer‐specific mortality hazard ratio = 1.81, 95% CI, 1.06‐2.97; *P* = .031). In addition, over half (56%) of these cases experienced disease recurrence in the lymph nodes. Our findings suggest that Barrett's adenocarcinoma may be an aggressive phenotype of EGJ adenocarcinoma due to the potential risk of tumor spread through the complex lympho‐vascular network of the esophagus.

## Graphical abstract

In this review article, we discussed future therapeutic targets of adenocarcinoma of esophagogastric junction (EGJ) according to the four molecular subtypes by TCGA. We also referred to the current understanding of the similarity between Barrett's adenocarcinoma and cardiac carcinoma, and added our own analysis examining prognostic differences between those two tumors using a multicenter cohort in Japan.

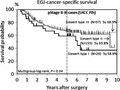

## 1 BARRETT'S AND CARDIAC ADENOCARCINOMA

There has been a gradual increase in the incidence of esophagogastric junction (EGJ) adenocarcinoma in Asian countries,^1^, ^2^, ^3^, ^4^ including Japan.^5^ Despite the recent advances in comprehensive genetic analyses as well as the progress in multimodal treatments, EGJ adenocarcinoma is still an aggressive malignancy with poor outcomes. EGJ adenocarcinoma includes Barrett's adenocarcinoma (also known as esophageal adenocarcinoma, or adenocarcinoma in distal esophagus) and cardiac adenocarcinoma (adenocarcinoma of gastric cardia) with esophageal invasion.^6^ Barrett's and cardiac adenocarcinomas have been increasing in parallel since the late 1970s in Western countries, and are recognized as common upper gastrointestinal cancers.^7^ There are numerous similarities between Barrett's and gastric adenocarcinomas in terms of tumor characteristics and background, with few differences highlighted to date.

In histological examinations, Barrett's adenocarcinoma and intestinal‐type gastric adenocarcinoma have a common premalignant background of intestinal metaplasia caused by chronic inflammation. Intestinal‐type gastric adenocarcinoma in the body and antrum of the stomach is mainly related to *Helicobacter pylori*‐induced chronic gastritis.^8^ *Helicobacter pylori* infection can also induce both chronic inflammation and metaplasia in the gastric cardia and cardiac adenocarcinoma.^9^, ^10^, ^11^, ^12^, ^13^ In addition, in terms of risk factors, both Barrett's and cardiac adenocarcinoma are associated with obesity in Western countries.^14^, ^15^, ^16^, ^17^ Adipokines produced from adipose tissue in association with metabolic syndrome can influence the development of chronic inflammation and cancer progression.^18^, ^19^ Finally, experimental data from transgenic mouse models suggest that Barrett's metaplasia may arise from gastric cardia progenitor cells in response to bile acid‐mediated inflammation via LGR5 expression and IL‐1β–IL‐6 signaling.^20^ Cholecystokinin 2 receptor (CCK2R, also known as CCKBR), which regulates gastric stem cells in the cardia or antrum regions of the stomach, is also upregulated in Barrett's esophagus and in esophageal adenocarcinoma.^21^, ^22^ Although a few studies have proposed the esophageal submucosal gland as the origin of Barrett's adenocarcinoma, considering previous perspective studies, Barrett's adenocarcinoma is likely to be of gastric origin.^23^, ^24^

## 2 CANDIDATE MOLECULES OF THERAPEUTIC TARGETS

Previously, gastroesophageal tumors were predominantly classified by pathological classification; however, in recent years — in the era of next‐generation sequencing technology — a molecular taxonomy has emerged.^25^, ^26^, ^27^, ^28^ The Cancer Genome Atlas (TCGA) Network has shown that gastroesophageal adenocarcinoma can be categorized into four molecular subtypes: Epstein‐Barr virus (EBV)‐associated, high‐level microsatellite instability (MSI‐H), genomically stable (GS), and chromosomal instability (CIN) tumors. These subtypes are classified using a range of techniques, including somatic copy number aberration, whole‐genome and whole‐exon sequencing, RNA sequencing, methylation assays, and proteomics analysis.^26^, ^28^ In the multiomic data of EGJ adenocarcinoma acquired from TCGA from cBioPortal for Cancer Genomics (https://www.cbioportal.org), there were a total of 172 cases which were classified as gastroesophageal junctional categories, including seven cases (4.1%) of MSI‐H, six cases (3.5%) of EBV, 11 cases (6.4%) of GS, and 148 cases (86%) of CIN.^26^, ^28^ In this section, we discuss the potential therapeutic targets of adenocarcinoma from esophagogastric junction (EGJ) as well as gastric adenocarcinoma, according to these four molecular subtypes.

High‐level microsatellite instability tumors (MSI‐H), which are not common in EGJ, harbor hypermutations, hypermethylations, *MLH1* silencing, immune reactivity, and demonstrate frequent mutations in various genes, including *ARID1A*, *RNF43*, *PIK3CA,* and *KRAS*.^5^, ^26^, ^28^ Based on the recent exploratory analysis of the Medical Research Council Adjuvant Gastric Infusional Chemotherapy (MAGIC) Trial, MSI‐H was considered a favorable prognostic factor, and chemotherapy was deemed not beneficial to patients with operable MSI‐H gastroesophageal tumors. Hence, surgery alone would be sufficient to treat MSI‐H cases.^29^ Although the MAGIC trial did not include any MSI‐H EGJ tumors, we have previously reported MSI‐H was detected in 7.6% of Siewert type II (11 cases of 145 patients), and 16.7% in Siewert type III EGJ adenocarcinoma (four cases of 24 patients).^5^ Tumors with hypermutations tend to produce neoantigens in the tumor microenvironment; thus, MSI‐H tumors are commonly immunogenic, and the host will often activate anti‐tumor immunity against these neoantigens.^30^ In addition, MSI‐H tumors can upregulate immunological checkpoints such as programmed death receptor‐1 (PD‐1), programmed death ligand‐1 (PD‐L1), or PD‐L2, in order to escape the host anti‐tumor immunity. In a randomized controlled clinical trial, Nivolumab treatment was shown to exert a significant survival benefit to patients with metastatic gastric or EGJ adenocarcinoma.^31^, ^32^ Nivolumab is an immune checkpoint inhibitor that blocks PD‐1. It may therefore be more effective for an MSI‐H population.

Epstein‐Barr virus‐associated tumors show hypermethylation, *CDKN2A* silencing, frequent mutations in *PIK3CA* and *ARID1A*, and immune reactivity.^26^, ^28^ EBV‐associated tumors seem to occupy only a small fraction of EGJ adenocarcinoma in TCGA data. Drugs that inhibit the PI3K pathway or methylation may be potentially beneficial for these tumors. Recent comprehensive genomic analyses revealed a significant mRNA expression of PD‐L1 and PD‐L2 in EBV‐associated gastric adenocarcinoma, suggesting that, like MSI‐H tumors, these tumors are also sensitive to immune checkpoint inhibitors, such as Nivolumab.^28^, ^31^

Genomically stable tumors are associated with diffuse histology, and present with frequent mutations in *CDH1* and *RHOA*, and the *CLDN18‐ARHGAP26* fusion gene.^26^, ^28^ Besides these major alterations, mutations in *BRCA1*, *BRCA2*, *RAD51C*, *PALB2*, and *CTNNA1* are detected in diffuse‐type gastric adenocarcinoma.^33^, ^34^, ^35^, ^36^ A proteomics analysis of 84 cases of diffuse gastric adenocarcinoma proposed classification according to the following three groups: PX1, which describes tumors expressing cell‐cycle dysregulated proteins; PX2, which describes tumors expressing epithelial‐mesenchymal transition pathway proteins as well as cell‐cycle‐related proteins; and PX3, which describes tumors with an enrichment of immunological proteins. Of note, tumors in the PX3 group have overexpressed IDO1 and ARG1 immunosuppressive proteins, for which inhibitory agents are already actionable.^37^

Finally, CIN tumors are described as having structural chromosomal aneuploidy without hypermutation, frequent *TP53* mutations, whole‐genome doubling, and amplification of cell‐cycle genes and genes from the receptor tyrosine kinase (RTK)‐RAS signaling pathway.^26^, ^28^, ^38^, ^39^ Because the CIN subtype dominates EGJ adenocarcinoma as described earlier, an understanding of the characteristics of this tumor subtype is crucial to improve therapeutic outcomes for patients with EGJ adenocarcinoma. Liu et al described two novel CIN subtypes, designated by scoring the quantity and intensity of focal, high‐level amplicons and using a combined analysis of TCGA data, with 921 cases of gastrointestinal adenocarcinoma: CIN‐Focal (CIN‐F), defined as tumors with high‐amplitude focal amplicons; and CIN‐Broad (CIN‐B), defined as tumors with low‐amplitude, broad amplicons.^39^ The authors reported that 74% of upper GI adenocarcinoma cases displayed CIN‐F. CIN‐F tumors frequently harbor mutations in *TP53*, and demonstrate amplifications in cell‐cycle‐related genes and RTK‐related pathway components, such as *KRAS*. We recently described a novel therapeutic strategy for *KRAS*‐amplified tumors.^40^ We found that *KRAS*‐amplified gastric cancer cells showed overexpression of KRAS protein, possessing a large pool of inactive KRAS (KRAS‐GDP state). *KRAS*‐amplified tumor cells show insensitivity to MAPK blockade as they can adaptively respond by mobilization of their reserve inactive KRAS to increase KRAS‐GTP state. Such adaptive responses can be abrogated through inhibition of the guanine‐exchange factors SOS1 and SOS2 or the protein tyrosine phosphatase SHP2, which can lead to inhibition of tumor growth when combined with MEK blockade. In TCGA dataset, *KRAS*‐amplified tumor occupied 8.1% of CIN‐type EGJ adenocarcinoma and 11.3% of CIN‐type gastric adenocarcinoma, which were more frequently observed as compared to the other types of cancers (5.7% in non‐small cell lung adenocarcinoma, 4.4% in pancreatic adenocarcinoma, 3.7% in bladder urothelial carcinoma, 1.9% in uterine corpus endometrial carcinoma, 1.4% in breast invasive carcinoma, 1.0% in colorectal adenocarcinoma, 0.8% in liver hepatocellular carcinoma, and 0.6% in prostate adenocarcinoma).^26^, ^28^, ^41^ Thus, combined inhibition of MEK and SHP2 may be one of the promising therapeutic approaches for CIN‐type EGJ adenocarcinoma as well as gastric adenocarcinoma.

Considering a recent emergence of nivolumab or pembrolizumab, PD‐L1 status is important molecular information in EGJ adenocarcinoma as well as gastric adenocarcinoma. There are several studies examining PD‐L1 positivity of EGJ adenocarcinoma separated from that of gastric adenocarcinoma (Table 1).^42^, ^43^, ^44^, ^45^, ^46^, ^47^, ^48^ PD‐L1 positivity of EGJ adenocarcinoma seems to be similar to that of gastric adenocarcinoma, despite different definitions of PD‐L1 positivity by immunohistochemical staining across studies.^42^, ^43^, ^44^ Focusing on EGJ adenocarcinoma, tumor PD‐L1 expression according to Siewert classification were conflicting across the studies.^45^, ^46^, ^48^ Further study is needed to address whether PD‐L1 status differs according to tumor location in EGJ adenocarcinoma.^45^, ^46^, ^47^, ^48^

**Table 1:** Previous studies examining PD‐L1 expression of EGJ adenocarcinoma separated from that of gastric adenocarcinoma

No.	Ref.	Year	Author (Country)		EGJ/esophagus or gastric (No. of cases)	Method	Tumor PD‐L1 positivity (%)		Stromal PD‐L1 positivity	Definition of PD‐L1 positivity
1	^41^	2018	Weinberg et al (USA)		EGJ (N = 119) Gastric (N = 462)	IHC	EGJ, 9.7% Gastric, 7.6%		No data	Tumor, ≥5% tumor cell membranous expression
2	^42^	2017	Xing et al (China)		EGJ (N = 8) Gastric (N = 4)	IHC	EGJ, 66.7% Gastric, 58.3%		EGJ, 75% Gastric, 50%	≥1% tumor cell expression Stroma, any immune cell expression (≥1%)
3	^43^	2015	Thompson et al (USA)		EGJ (N = 5) Gastric (N = 29)	IHC	EGJ, 0% Gastric, 13.8%		EGJ, 20% Gastric, 48.3%	Tumor, ≥5% tumor cell expression Stroma, any immune cell expression (≥1%)
4	^44^	2015	Derks et al (USA)		EGJ/esophagus (N = 344)	IHC	EGJ/esophagus, 18% (mid‐proximal esophagus, 0.6% distal esophagus, 4.7% EGJ, 18.3%)		No data	Tumor, ≥5% tumor cell membranous expression (by tumor tissue microarray)
5	^45^	2018	Kollmann et al (Austria, Czech, Switzerland and USA)		EGJ/esophagus (N = 168)	IHC	EGJ/esophagus, 18% (Siewert type I, 26.8%; type II, 8.3%; type III, 8.3%)		No data	Tumor, any tumor cell expression (≥1%)
6	^46^	2019	Knief et al (Germany)	EGJ/esophagus (N = 135)		IHC	EGJ/esophagus, 48.1%			Combined positive score ≥1%
7	^47^	2020	Wang et al (China)	EGJ/esophagus (N = 96)		IHC	EGJ/esophagus, 11.5% (Siewert type I, 0%; type II, 7.9%; type III, 15.7%)	No data		Tumor, ≥5 stained tumor cells in a 400x field

Abbreviations: EGJ, esophagogastric junction; IHC, immunohistochemistry.

Beyond TCGA molecular subtypes, the Asian Cancer Research Group (ACRG) has also categorized gastroesophageal cancers, and proposed four subtypes based solely on gene expression signatures: MSI, MSS/epithelial‐to‐mesenchymal transition (EMT), MSS/TP53‐active, and MSS/TP53‐deficient tumors.^49^ The MSS/TP53‐deficient subtype, which is enriched with TCGA CIN subtype due to substantial aneuploidy, is frequently observed in the EGJ cardiac adenocarcinoma. The ACRG study also performed a survival analysis comparing their subtyping scheme with that of TCGA subtyping. Under the ACRG subtyping scheme, the MSI cases showed the most favorable outcomes, followed by MSS/TP53‐active, MSS/TP53‐inactive, and MSS‐EMT. When classified by TCGA subtyping, MSI cases still showed the best outcomes, but there were no significant prognostic differences among the EBV, GS, and CIN subtypes. This discrepancy may be because the disease stage was biased across the molecular subtypes in both studies. Further analyses are needed to assess how molecular subtype confers prognostic impact in gastroesophageal adenocarcinoma.

## 3 CLINICAL AND PROGNOSTIC DIFFERENCES BY TUMOR LOCATION

Esophagogastric junction adenocarcinoma extends across the thorax and abdomen to varying degrees. For patients with EGJ adenocarcinoma, tumor location is specified by Siewert classification as follows: type I, defined as tumors of the distal esophagus, in which the epicenter of the tumor is located 1‐5 cm above the anatomical EGJ; type II, true junctional tumors, in which the epicenter is located 1 cm above and 2 cm below the EGJ; and type III, gastric tumors that infiltrate into the esophagus, for which the epicenter is located between 2 and 5 cm below the EGJ.^50^ Although there does not seem to be any difference in terms of the carcinogenic origin of Barrett's and cardiac adenocarcinoma, as mentioned earlier, this anatomical classification may differentiate these two tumors in terms of clinicopathological or prognostic characteristics. Considering that Barrett's esophagus involves a replacement of normal squamous epithelium with metaplastic mucosa in response to gastroesophageal reflux disease (GERD), Barrett's adenocarcinoma is likely to be located more proximally (Siewert type I) than cardiac adenocarcinoma.^51^ Furthermore, because tumor cells of EGJ adenocarcinoma can spread in various longitudinal pathways through the complex lympho‐vascular network in the submucosal layer, Siewert classification is likely to be associated with tumor aggressiveness.^52^, ^53^ Indeed, Siewert classification is frequently employed to determine a surgical approach: patients with Siewert type I tumors are often treated through a thoracic approach with mediastinal lymph node dissection, whereas patients with Siewert type II or III cases can be treated via a transabdominal approach (so‐called “transhiatal” approach) when technically possible.

A number of studies have examined the clinicopathological and prognostic characteristics of EGJ adenocarcinoma via a comparative analysis of Siewert type I‐III tumors.^54^, ^55^, ^56^, ^57^, ^58^, ^59^, ^60^ However, the associations between Siewert type and surgical outcome are conflicting: Siewert type III tumors appear to have a worse clinical outcome,^56^, ^61^ but this difference is not significant when differentiated by tumor location.^54^, ^55^, ^57^ Here, we sought to investigate the clinical features and prognostic outcomes of EGJ adenocarcinoma, using 395 patients with Siewert type I‐III tumors who underwent surgical resection without neoadjuvant chemotherapy or radiotherapy in Japan.

### 3.1 Patient cohort of EGJ adenocarcinoma

**FIGURE 1:**
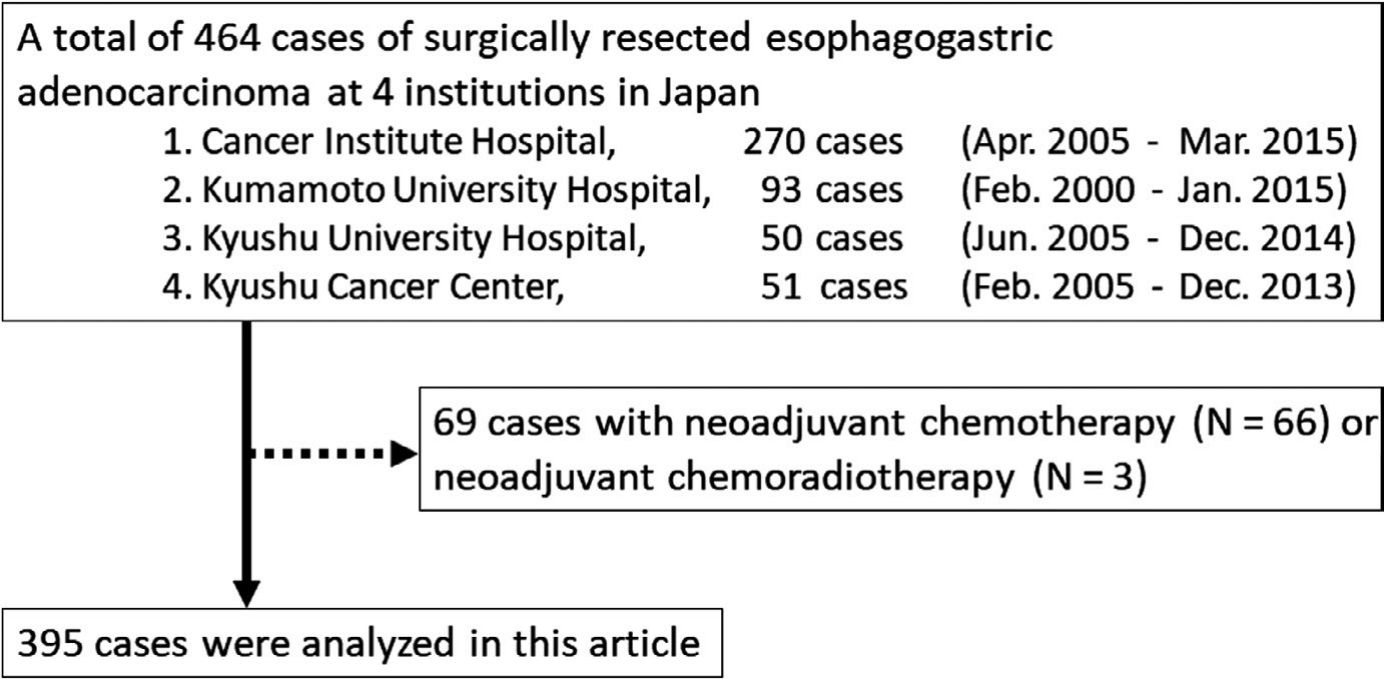
Flow diagram of our multicenter retrospective cohort of esophagogastric adenocarcinoma

Our multicenter retrospective cohort included 464 patients with EGJ adenocarcinoma (Siewert type I, II, and III) who underwent surgical resection at four academic institutions in Japan between February 2000 and March 2015 (Figure 1). A total of 395 patients with EGJ adenocarcinoma were eligible for this study. Disease staging was based on the 7th edition of the Union for International Cancer Control (UICC) classification of esophageal cancer, which is applicable to Siewert type III tumors.

### 3.2 Statistical analysis

All statistical analyses were performed using JMP 13 software (Version 13.2.1, SAS Institute). All *P*‐values were two‐sided. Univariate analyses were performed to investigate clinicopathological and molecular characteristics according to Siewert classification. Chi‐squared test or Fisher's exact test was used for categorical data, whereas the Wilcoxon test or Kruskal‐Wallis test was used for continuous data. The Kaplan‐Meier method was used to estimate survival distribution, and the log‐rank test was used to compare survival distributions. EGJ‐cancer‐specific survival time for each case was calculated from the date of surgical resection until death from EGJ adenocarcinoma, or 10 March 2020, whichever came first. Relapse‐free survival (RFS) time was from the date of surgical resection until recurrence or death from any cause, and overall survival (OS) until death from any cause. Cox proportional hazards models were used to estimate mortality hazard ratios (HRs). More details are provided in Appendix S1.

### 3.3 Clinicopathological and survival analysis in terms of tumor location

The baseline characteristics according to Siewert classification are shown in Table 2. Type I tumors were observed in 59 cases (15%), type II in 280 cases (71%), and type III in 56 cases (14%). Type I tumors were associated with recent cases (*P* < .0001), more frequently accompanied by Barrett's esophagus (78%, *P* < .0001), less frequently associated with *Helicobacter pylori* infection (*P* < .0001), and showed a less‐advanced disease stage as compared with type II or III tumors (*P* = .0002). Because surgeons often select a transthoracic approach for type I tumors (*P* < .0001), operative time was the longest for type I tumors (*P* < .0001), with less blood loss and a successful R0 resection rate (*P* = .0041). Patients with type III tumors exhibited a significantly larger size (*P* < .0001), because the radius of the tumor in type III tumors must be at least 2 cm to invade into the esophagus. Accordingly, type III cases included more advanced disease stage, longer operative time with more blood loss, and a lower R0 resection (Table 2).

**Table 2:** Baseline characteristics according to Siewert classification (N = 395)

Clinicopathological features	Total No.	Tumor location by Siewert classification			*P* (across 3 Siewert types)
		Type I (N = 59)	Type II (N = 280)	Type III (N = 56)	
Sex					
Female	63 (16%)	5 (9%)	46 (16%)	12 (21%)	.15
Male	332 (84%)	54 (91%)	234 (84%)	44 (79%)	
Age at surgery (y, mean ± SD)	65.0 ± 12.3	63.1 ± 11.5	64.7 ± 12.6	68.5 ± 11.3	.060
Year since surgery					
Before Dec. 2009	181 (46%)	15 (25%)	128 (46%)	38 (68%)	**<.0001**
Jan. 2010 to Mar. 2015	214 (54%)	44 (75%)	152 (54%)	18 (32%)	
Body mass index (kg/m^2^, mean ± SD)	22.7 ± 3.4	23.1 ± 4.3	22.8 ± 3.2	22.1 ± 3.4	.28
<22.6 (median)	195 (50%)	25 (43%)	138 (50%)	32 (57%)	.33
≥22.6 (median)	196 (50%)	33 (57%)	139 (50%)	24 (43%)	
Tumor diameter (mm, mean ± SD)	54.9 ± 30.7	45.1 ± 28.0 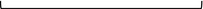	51.4 ± 28.1 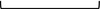	82.5 ± 31.1	**<.0001**
<50 (median)	185 (47%)	39 (66%)	139 (50%)	7 (13%)	**<.0001**
≥50 (median)	210 (53%)	20 (34%)	141 (50%)	49 (87%)	
Barrett's esophagus					
Absent	271 (69%)	13 (22%)	204 (73%)	54 (96%)	**<.0001**
Present	124 (31%)	46 (78%)	76 (27%)	2 (4%)	
*Helicobacter pylori* infection (Limited to Cancer Institute Hospital cases)					
Negative	100 (44%)	35 (79%)	64 (39%)	1 (5%)	**<.0001**
Positive	128 (56%)	9 (21%)	100 (61%)	19 (95%)	
pT Stage					
pT1	101 (26%)	27 (46%)	71 (25%)	3 (5%)	**<.0001**
pT2	54 (14%)	6 (10%)	47 (17%)	1 (2%)	
pT3	137 (34%)	23 (39%)	95 (34%)	19 (34%)	
pT4	103 (26%)	3 (5%)	67 (24%)	33 (59%)	
pN Stage					
pN0	157 (39%)	28 (47%)	117 (41%)	12 (21%)	**.021**
pN1	79 (20%)	10 (17%)	58 (21%)	11 (20%)	
pN2	65 (17%)	8 (14%)	47 (17%)	10 (18%)	
pN3	94 (24%)	13 (22%)	58 (21%)	23 (41%)	
No. of nodes harvested (mean ± SD)	34.9 ± 17.8	37.3 ± 19.8	33.3 ± 16.1 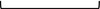	40.6 ± 22.0	**.020**
No. of metastatic nodes (mean ± SD)	4.2 ± 6.7	3.5 ± 5.3 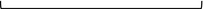	3.7 ± 5.9 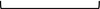	7.7 ± 9.9	**.0010**
M stage					
M0	336 (85%)	54 (91%)	242 (86%)	40 (71%)	**.0051**
M1	59 (15%)	5 (9%)	38 (14%)	16 (29%)	
pStage					
I	115 (29%)	25 (42%)	87 (31%)	3 (5%)	**.0002**
II	51 (13%)	7 (12%)	37 (13%)	7 (13%)	
III	170 (43%)	22 (37%)	118 (42%)	30 (53%)	
IV	59 (15%)	5 (9%)	38 (14%)	16 (29%)	
Adjuvant chemotherapy					
Absent	237 (61%)	35 (59%)	176 (63%)	26 (48%)	.10
Present	153 (39%)	24 (41%)	101 (37%)	28 (52%)	
Histological subtypes					
Lauren classification					
Intestinal	305 (77%)	49 (83%)	217 (77%)	39 (70%)	.23
Diffuse	90 (23%)	10 (17%)	63 (23%)	17 (30%)	
WHO classification					
Papillary	7 (2%)	1 (2%)	4 (1%)	2 (4%)	.30
Tubular	280 (70%)	47 (79%)	200 (71%)	33 (59%)	
Mucinous	18 (5%)	1 (2%)	13 (5%)	4 (7%)	
Poorly cohesive	90 (23%)	10 (17%)	63 (23%)	17 (30%)	
Lymphatic invasion					
Absent	123 (32%)	26 (44%)	82 (30%)	15 (27%)	.077
Present	265 (68%)	33 (56%)	191 (70%)	41 (73%)	
Venous invasion					
Absent	130 (33%)	27 (46%)	84 (30%)	19 (34%)	.076
Present	261 (67%)	32 (54%)	192 (70%)	37 (66%)	
Surgical approach					
Transhiatal	313 (79%)	13 (22%)	244 (87%)	56 (100%)	**<.0001**
Transthoracic	82 (21%)	46 (78%)	36 (13%)	0	
Operative time (min, mean ± SD)	346 ± 141	493 ± 149 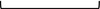 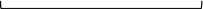	321 ± 123	318 ± 127	**<.0001**
<320 (median)	194 (50%)	5 (9%)	158 (57%)	31 (56%)	**<.0001**
≥320 (median)	194 (50%)	52 (91%)	118 (43%)	24 (44%)	
Blood loss volume (g, mean ± SD)	461 ± 414	450 ± 400 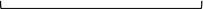	432 ± 391 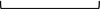	613 ± 502	**.0084**
<350 (median)	188 (49%)	28 (49%)	139 (50%)	21 (38%)	.25
≥350 (median)	199 (51%)	29 (51%)	136 (50%)	34 (62%)	
Blood transfusion					
Absent	343 (88%)	51 (89%)	248 (90%)	44 (79%)	.056
Present	46 (12%)	6 (11%)	28 (10%)	12 (21%)	
Resection margin					
R0	346 (87%)	57 (96%)	248 (89%)	41 (73%)	**.0041**
R1	26 (7%)	1 (2%)	17 (6%)	8 (14%)	
R2	23 (6%)	1 (2%)	15 (5%)	7 (13%)	
Preoperative complications					
None or Clavien‐Dindo < IIIa	315 (80%)	41 (69%)	227 (81%)	47 (84%)	.093
Clavien‐Dindo ≥ IIIa	80 (20%)	18 (31%)	53 (19%)	9 (16%)	

Note

(%) Indicates the proportion of cases with specific clinicopathological features for each Siewert classification group. ^*^ *P* < .05, **P < .01, ***P < .001.

Abbreviation: SD, standard deviation.

**FIGURE 2:**
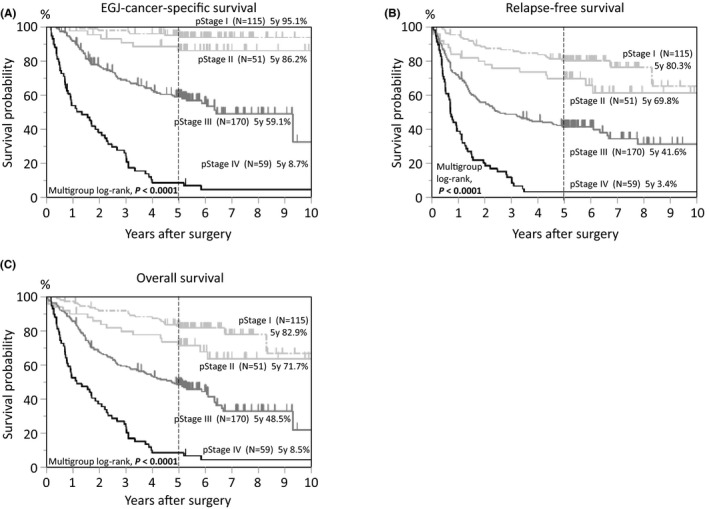
Kaplan‐Meier curves of 395 cases of esophagogastric junction (EGJ) adenocarcinoma, categorized according to disease stage (pStage I‐IV). A, EGJ‐cancer‐specific survival. B, Relapse‐free survival. C, Overall survival

**FIGURE 3:**
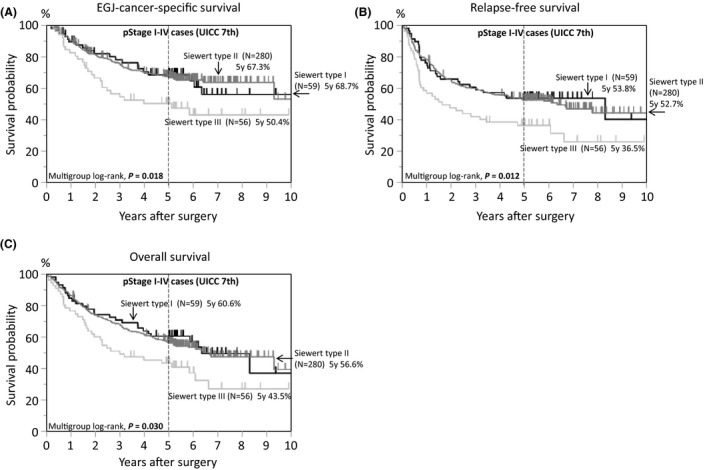
Kaplan‐Meier curves of 395 cases of esophagogastric junction (EGJ) adenocarcinoma according to tumor location by Siewert classification. A, EGJ‐cancer‐specific survival. B, Relapse‐free survival. C, Overall survival

**FIGURE 4:**
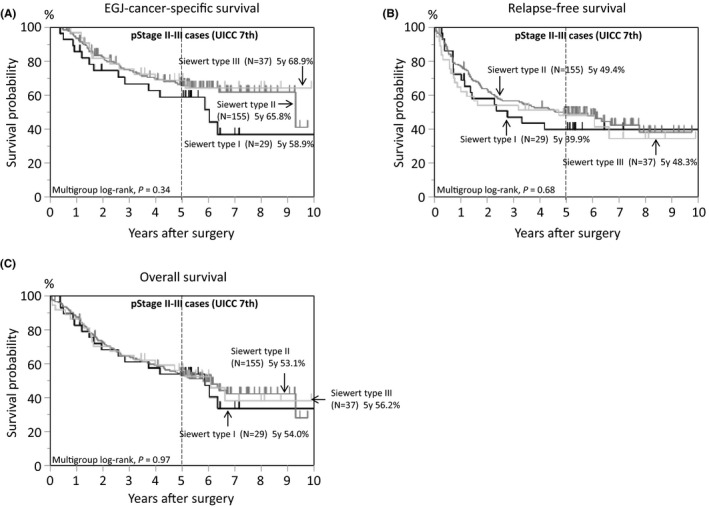
Kaplan‐Meier curves of pStage II‐III cases (N = 221) of esophagogastric junction (EGJ) adenocarcinoma according to tumor location by Siewert classification. A, EGJ‐cancer‐specific survival. B, Relapse‐free survival. C, Overall survival

In the survival analysis, there were 192 deaths, including 135 EGJ‐cancer‐specific deaths, over a median follow‐up of 5.3 years (interquartile range, 5.0‐6.6 years) for censored cases. Kaplan‐Meier analyses according to disease stage are provided in Figure 2A‐C. The 5‐year EGJ‐cancer‐specific survival rates were 95.1% for pStage I, 86.2% for pStage II, 59.1% for pStage III, and 8.7% for pStage IV (Figure 2A). Kaplan‐Meier analyses according to Siewert classification (N = 395, Figure 3A‐C) showed that the 5‐year EGJ‐cancer‐specific mortality of Siewert type III tumors was the worst (50.4%, Figure 3A) compared with that of type I (68.7%) or type II (67.3%) tumors. This result is consistent with previous reports, due to the bias of more advanced disease cases among type III tumors.^47^, ^52^ When adjusting for various clinical factors in the multivariate survival analysis, the significant dismal prognosis of type I tumors became evident using type II tumors as a reference (multivariate EGJ‐cancer‐specific mortality HR = 1.81, 95% confidence interval [CI], 1.06‐2.97; *P* = .031; Table 3). A subgroup analysis of pStage II‐III cases (N = 221, Figure 4A‐C) showed that the 5‐year EGJ‐cancer‐specific mortality of Siewert type I cases (58.9%, Figure 4A) was the highest as compared with that of type II (65.8%) or type III (68.9%) cases (multivariate mortality HR = 2.13, 95% confidence interval [CI], 1.09‐3.98; *P* = .028; N = 221, Table 4). Regarding recurrence among type I tumors after surgery, over half of type I cases (56%) experienced recurrent disease in lymph nodes, particularly in mediastinal or paraaortic nodes (Table S1). This raises a clinically important question, whether radical lymph node dissection, including removal of mediastinal or paraaortic node dissection, may be beneficial or not for patients with Siewert type I tumors. Further large‐scale study would be needed to address this issue.

**Table 3:** Patient mortality in all cases (N = 395)

Clinicopathological factors	Total No.	EGJ‐cancer‐specific survival			Relapse‐free survival			Overall survival		
		No. of events	Univariate HR (95% CI)	Multivariate HR (95% CI)	No. of events	Univariate HR (95% CI)	Multivariate HR (95% CI)	No. of events	Univariate HR (95% CI)	Multivariate HR (95% CI)
Siewert classification										
Type I (vs Type II)	59	20	1.02 (0.61‐1.62) *P* = .94	1.81 (1.06‐2.97) ***P* = .031**	28	0.96 (0.62‐1.41) *P* = .83	1.35 (0.87‐2.03) *P* = .18	27	0.93 (0.60‐1.38) *P* = .72	1.43 (0.91‐2.18) *P* = .12
Type III (vs Type II)	56	27	1.83 (1.17‐2.78) ***P* = .0094**	0.93 (0.59‐1.44) *P* = .76	37	1.69 (1.16‐2.41) ***P* = .0071**	0.95 (0.64‐1.38) *P* = .81	35	1.61 (1.09‐2.31) ***P* = .018**	0.92 (0.61‐1.33) *P* = .65

Note

The multivariate, Cox proportional hazard regression model initially included gender, age, year of surgery, body mass index, tumor diameter, tumor location by Siewert classification, existence of Barrett's esophagus, disease stage, tumor differentiation, lymphatic invasion, venous invasion, surgical approach, operative time, blood loss volume, blood transfusion, resection margin, preoperative complication, adjuvant chemotherapy.

A backward elimination with a threshold of *P* = .20 was used to select variables in the final model.

Abbreviations: CI, confidence interval; HR, hazard ratio.

**Table 4:** Patient mortality according to Siewert classification in pStage II and III cases (N = 221)

	Total No.	EGJ‐cancer‐specific survival			Relapse‐free survival			Overall survival		
		No. of events	Univariate HR (95% CI)	Multivariate HR (95% CI)	No. of events	Univariate HR (95% CI)	Multivariate HR (95% CI)	No. of events	Univariate HR (95% CI)	Multivariate HR (95% CI)
Tumor location (vs Siewert type II)										
Siewert type I	29	14	1.51 (0.80‐2.67) *P* = .19	**2.13 (1.09‐3.98)** ***P* = .028**	17	1.20 (0.69‐1.98) *P* = .50	1.64 (0.92‐2.78) *P* = .093	16	1.08 (0.61‐1.79) *P* = .79	1.57 (0.86‐2.70) *P* = .14
Siewert type III	37	11	0.93 (0.46‐1.73) *P* = .84	0.90 (0.44‐1.68) *P* = .75	21	1.18 (0.71‐1.86) *P* = .52	1.12 (0.67‐1.77) *P* = .66	19	1.01 (0.59‐1.62) *P* = .98	0.95 (0.56‐1.54) *P* = .85

Note

The multivariate Cox regression model included the same set of covariates selected as in Table 2.

Abbreviations: CI, confidence interval; HR, hazard ratio.

Because Siewert type I tumors were significantly associated with Barrett's esophagus, additional analyses were performed focusing on the presence or absence of Barrett's esophagus. Although the clinicopathological features of the cases accompanied by Barrett's esophagus was similar to those of Siewert type I tumors (Tables S2 and S3), there was no significant association between Barrett's esophagus and patient outcome in multivariate survival analysis (Tables S4 and S5).

## 4 CONCLUSION

Recent comprehensive genomic analyses suggest that immunotherapy may play an essential role in the treatment of MSI‐H, EBV, or GS molecular subtypes of EGJ adenocarcinoma. EGJ adenocarcinoma includes Barrett's adenocarcinoma and cardiac adenocarcinoma, and because of their extensive similarities, it is likely that Barrett's adenocarcinoma is of gastric origin. In our multicenter cohort study, we show that Siewert type I (distal esophagus) tumors are frequently accompanied with Barrett's esophagus and have significantly unfavorable outcomes, with more than half of patients experiencing disease recurrence in the lymph nodes. Thus, Barrett's adenocarcinoma may potentially be an aggressive clinical subtype of EGJ adenocarcinoma, with a potential risk of tumor spreading through the complex lympho‐vascular network of the esophagus.

## CONFLICT OF INTERESTS

None of the authors has any conflict of interest related to this study.

## Supporting information

Appendix S1

Table S1‐S5
