# Supplementary material for: Esophagogastric junction adenocarcinoma shares characteristics with gastric adenocarcinoma: Literature review and retrospective multicenter cohort study
Source: Ann Gastroenterol Surg. 2020 Oct 26;5(1):46–59. doi: 10.1002/ags3.12406 (PMC7832959; doi:10.1002/ags3.12406)
Supplement: Supplementary file 1 — Appendix S1 [file AGS3-5-46-s001.docx]

**Supporting information**

**APPENDIX**

Histopathological evaluations were carried out by two pathologists who were blinded to the other data. Histological type was categorized by Lauren’s criteria and WHO classification based on the dominant histological type.^1^ The presence of Barrett’s esophagus was diagnosed by endoscopists or pathologists. Limited to the cases from the Cancer Institute Hospital, *H. pylori* infection status was diagnosed by an endoscopist as the presence or absence of a regular arrangement of collecting venules or atrophic gastritis.^2^

The multivariate model initially included the following clinicopathological and molecular variables: sex (male vs. female), age (as determined by a median split, <66 vs. ≥66), year of surgery (median split, before December 2009 vs. after January 2010), body mass index (BMI, median split, <22.6 vs. ≥22.6 kg/m^2^), tumor macroscopic diameter in specimen (median split, <50 vs. ≥50 mm), tumor location according to Siewert classification (type I vs. II vs. III), coexistence of Barrett’s esophagus (presence vs. absence), disease stage according to the 7th AJCC/UICC TNM classification of esophageal cancer (I vs. II vs. III vs. IV), dominant histological type by Lauren’s classification (diffuse vs. intestinal type), lymphatic invasion (presence vs. absence), venous invasion (presence vs. absence), surgical approach (transthoracic vs. transhiatal), operative time (median split, <320 vs. ≥320 min), blood loss volume (median split, <350 vs. ≥350 g), blood transfusion (presence vs. absence), resection margin (R0 vs. R1 vs. R2), preoperative complications according to Clavien-Dindo classification (none or I-IIIa vs. IIIa-V), and adjuvant chemotherapy (presence vs. absence). A backward elimination was performed with a threshold of *P* = 0.10 to avoid overfitting. Less than 10% of patients had missing information. Cases with missing information for any of the categorical covariates (BMI [1.0%], lymphatic invasion [1.8%], venous invasion [1.0%], operative time [1.8%], blood loss [2.0%], blood transfusion [1.5%], and adjuvant chemotherapy [1.3%]), were treated using the informative missing option of JMP 13 software. We confirmed that excluding cases (i.e., those missing information in any of the covariates) did not substantially alter the results (data not shown).

Institutional Review Board numbers are as follows: 2015-1107 for Cancer Institute Hospital, 858 for Kumamoto University Hospital, 2015-26 and 2020-6 for Kyushu Cancer Center, and 820-00 for Kyushu University Hospital.

**REFERENCES FOR APPENDIX**

1. Lauren P. The Two Histological Main Types of Gastric Carcinoma: Diffuse and So-Called Intestinal-Type Carcinoma. An Attempt at a Histo-Clinical Classification. Acta Pathol Microbiol Scand. 1965; 64: 31-49.

2. Yagi K, Aruga Y, Nakamura A, et al. Regular arrangement of collecting venules (RAC): a characteristic endoscopic feature of Helicobacter pylori-negative normal stomach and its relationship with esophago-gastric adenocarcinoma. J Gastroenterol. 2005; 40: 443-52.

**LIST OF SUPPLEMENTARY TABLE TITLES**

**SUPPLEMENTARY TABLE 1 Recurrent sites of pStage II-III cases according to Siewert classification (N = 221)**

**SUPPLEMENTARY TABLE 2 Baseline characteristics according to the presence or absence of Barrett’s esophagus (N = 395)**

**SUPPLEMENTARY TABLE 3 Recurrent sites of pStage II-III cases according to the presence or absence of Barrett’s esophagus (N = 221)**

**SUPPLEMENTARY TABLE 4 Mortality of the patient with Barrett’s esophagus** **in all cases (N = 395)**

**SUPPLEMENTARY TABLE 5 Mortality of the patient with Barrett’s esophagus in pStage II and III cases (N = 221)**
