# Supplementary material for: Esophagogastric junction adenocarcinoma shares characteristics with gastric adenocarcinoma: Literature review and retrospective multicenter cohort study
Source: Ann Gastroenterol Surg. 2020 Oct 26;5(1):46–59. doi: 10.1002/ags3.12406 (PMC7832959; doi:10.1002/ags3.12406)
Supplement: Supplementary file 2 — Table S1‐S5 [file AGS3-5-46-s002.docx]

**SUPPLEMENTARY TABLE 1 Recurrent sites of pStage II-III cases according to Siewert classification (N=221)**

| Clinicopathological features | Total No. | Tumor location by Siewert classification | | |
| --- | --- | --- | --- | --- |
|  |  | Type I  (N = 29) | Type II  (N = 155) | Type III  (N = 37) |
| Recurrence in pStage II-III cases |  |  |  |  |
| Absent | 138 (62%) | 13 (45%) | 101 (65%) | 24 (65%) |
| Present | 83 (38%) | 16 (55%) | 54 (35%) | 13 (35%) |
|  |  |  |  |  |
| The first site of recurrence (R0-1) |  |  |  |  |
| Liver | 22 (27%) | 2 (13%) | 16 (30%) | 4 (31%) |
| Peritoneum | 18 (22%) | 2 (13%) | 14 (26%) | 2 (15%) |
| Lung | 12 (15%) | 4 (25%) | 8 (15%) | 0 |
| Bone | 3 (4%) | 0 | 3 (6%) | 0 |
| Adrenal | 2 (2%) | 0 | 2 (4%) | 0 |
| Brain | 1 (1%) | 0 | 0 | 1 (8%) |
| Anastomosis | 7 (8%) | 0 | 5 (9%) | 2 (15%) |
| Pleural | 2 (2%) | 1 (6%) | 1 (2%) | 0 |
| Other | 1 (1%) | 0 | 1 (2%) | 0 |
| Lymph node (LN) | 31 (37%) | 9 (56%) | 19 (35%) | 3 (23%) |
| Cervical | 4 | 0 | 2 | 2 |
| Mediastinal | 13 | 3 | 10 | 0 |
| Para-aortic | 14 | 4 | 8 | 2 |
| Abdominal | 6 | 3 | 2 | 1 |
| Unknown | 0 | 0 | 0 | 0 |

(%) indicates the proportion of cases among each Siewert classification group.

**SUPPLEMENTARY TABLE 2 Baseline characteristics according to the presence or absence of Barrett’s esophagus (N = 395)**

| Clinicopathological features | Total No. | Barrett’s esophagus | | *P* |
| --- | --- | --- | --- | --- |
|  |  | Present  (N = 124) | Absent  (N = 271) |  |
| Sex |  |  |  | **0.045** |
| Female | 63 (16%) | 13 (11%) | 50 (19%) |  |
| Male | 332 (84%) | 111 (89%) | 221 (81%) |  |
|  |  |  |  |  |
| Age at surgery (y, mean ± SD) | 65.0±12.3 | 62.9±11.1 | 65.9±12.8 | **0.0084** |
|  |  |  |  |  |
| Year since surgery |  |  |  | **0.0001** |
| Before Dec. 2009 | 181 (46%) | 39 (32%) | 142 (52%) |  |
| Jan. 2010 to Mar. 2015 | 214 (54%) | 85 (69%) | 129 (48%) |  |
|  |  |  |  |  |
| Body mass index (kg/m^2^, mean ± SD) | 22.7±3.4 | 23.3±3.2 | 22.4±3.5 | **0.0027** |
| <22.6 (median) | 195 (50%) | 47 (38%) | 148 (55%) | **0.0018** |
| ≥22.6 (median) | 196 (50%) | 76 (62%) | 120 (45%) |  |
|  |  |  |  |  |
| Tumor diameter (mm, mean ± SD) | 54.9±30.7 | 37.7±23.2 | 62.7±30.5 | **<0.0001** |
| <50 (median) | 185 (47%) | 95 (77%) | 90 (33%) | **<0.0001** |
| ≥50 (median) | 210 (53%) | 29 (23%) | 181 (67%) |  |
|  |  |  |  |  |
| Tumor location  by Siewert classification |  |  |  | **<0.0001** |
| Type I | 59 (15%) | 46 (37%) | 13 ( 5%) |  |
| Type II | 280 (71%) | 76 (61%) | 204 (75%) |  |
| Type III | 56 (14%) | 2 ( 2%) | 54 (20%) |  |
|  |  |  |  |  |
| *Helicobacter pylori* infection  (Limited to Cancer Institute Hospital cases) |  |  |  | **<0.0001** |
| Negative | 100 (44%) | 62 (74%) | 38 (26%) |  |
| Positive | 128 (56%) | 22 (26%) | 106 (74%) |  |
|  |  |  |  |  |
| pT Stage |  |  |  | <0.0001 |
| pT1 | 101 (26%) | 64 (51%) | 37 (14%) |  |
| pT2 | 54 (14%) | 20 (16%) | 34 (13%) |  |
| pT3 | 137 (34%) | 33 (27%) | 104 (38%) |  |
| pT4 | 103 (26%) | 7 ( 6%) | 96 (35%) |  |
|  |  |  |  |  |
| pN Stage |  |  |  | **<0.0001** |
| pN0 | 160 (40%) | 71 (57%) | 89 (32%) |  |
| pN1 | 79 (20%) | 27 (21%) | 52 (19%) |  |
| pN2 | 65 (16%) | 13 (11%) | 52 (19%) |  |
| pN3 | 94 (24%) | 13 (11%) | 81 (30%) |  |
|  |  |  |  |  |
| No. of nodes harvested (mean ± SD) | 34.9±17.8 | 30.8±15.4 | 36.8±18.5 | **0.024** |
|  |  |  |  |  |
| No. of metastatic nodes (mean ± SD) | 4.2±6.7 | 2.1±4.8 | 5.2±7.2 | **<0.0001** |
|  |  |  |  |  |
| M stage |  |  |  | **0.0014** |
| M0 | 336 (85%) | 116 (93%) | 220 (81%) |  |
| M1 | 59 (15%) | 8 ( 7%) | 51 (19%) |  |
|  |  |  |  |  |
| pStage |  |  |  | **<0.0001** |
| I | 115 (29%) | 64 (51%) | 51 (19%) |  |
| II | 51 (13%) | 16 (13%) | 35 (13%) |  |
| III | 170 (43%) | 36 (29%) | 134 (49%) |  |
| IV | 59 (15%) | 8 ( 7%) | 51 (19%) |  |
|  |  |  |  |  |
| Adjuvant chemotherapy |  |  |  | **0.012** |
| Absent | 237 (61%) | 86 (70%) | 151 (57%) |  |
| Present | 153 (39%) | 37 (30%) | 116 (43%) |  |
|  |  |  |  |  |
| Histological subtypes |  |  |  |  |
| Lauren classification |  |  |  | **0.0080** |
| Intestinal | 305 (77%) | 106 (85%) | 199 (73%) |  |
| Diffuse | 90 (23%) | 18 (15%) | 72 (27%) |  |
|  |  |  |  |  |
| WHO classification |  |  |  | **0.019** |
| Papillary | 7 ( 2%) | 1 ( 1%) | 6 ( 2%) |  |
| Tubular | 280 (70%) | 101 (81%) | 179 (66%) |  |
| Mucinous | 18 ( 5%) | 4 ( 3%) | 14 ( 5%) |  |
| Poorly cohesive | 90 (23%) | 18 (15%) | 72 (27%) |  |
|  |  |  |  |  |
| Lymphatic invasion |  |  |  | **<0.0001** |
| Absent | 123 (32%) | 56 (46%) | 67 (25%) |  |
| Present | 265 (68%) | 65 (54%) | 200 (75%) |  |
|  |  |  |  |  |
| Venous invasion |  |  |  | **<0.0001** |
| Absent | 130 (33%) | 59 (48%) | 71 (27%) |  |
| Present | 261 (67%) | 64 (52%) | 197 (73%) |  |
|  |  |  |  |  |
| Surgical approach |  |  |  | **<0.0001** |
| Transhiatal | 313 (79%) | 75 (60%) | 238 (88%) |  |
| Transthoracic | 82 (21%) | 49 (40%) | 33 (12%) |  |
|  |  |  |  |  |
| Operative time (min, mean ± SD) | 346±141 | 405±161 | 319±123 | **<0.0001** |
| <320 (median) | 194 (50%) | 44 (36%) | 150 (56%) | **0.0002** |
| ≥320 (median) | 194 (50%) | 78 (64%) | 116 (44%) |  |
|  |  |  |  |  |
| Blood loss volume (g, mean ± SD) | 461±414 | 393±364 | 492±432 | **0.011** |
| <350 (median) | 188 (49%) | 67 (55%) | 121 (46%) | 0.091 |
| ≥350 (median) | 199 (51%) | 55 (45%) | 144 (54%) |  |
|  |  |  |  |  |
| Blood transfusion |  |  |  | 0.25 |
| Absent | 343 (88%) | 111 (91%) | 232 (87%) |  |
| Present | 46 (12%) | 11 ( 9%) | 35 (13%) |  |
|  |  |  |  |  |
| Resection margin |  |  |  | **0.0085** |
| R0 | 346 (87%) | 118 (96%) | 228 (84%) |  |
| R1 | 26 ( 7%) | 3 ( 2%) | 23 ( 9%) |  |
| R2 | 23 ( 6%) | 3 ( 2%) | 20 ( 7%) |  |
|  |  |  |  |  |
| Preoperative complications |  |  |  | 0.61 |
| None or Clavien-Dindo <IIIa | 315 (80%) | 97 (78%) | 218 (80%) |  |
| Clavien-Dindo ≥IIIa | 80 (20%) | 27 (22%) | 53 (20%) |  |

(%) indicates the proportion of cases with specific clinicopathological features for existence of Barrett’s esophagus.

SD, standard deviation.

**SUPPLEMENTARY TABLE 3 Recurrent sites of pStage II-III cases according to the presence or absence of Barrett’s esophagus (N=221)**

| Clinicopathological features | Total No. | Barrett’s esophagus | |
| --- | --- | --- | --- |
|  |  | Present  (N = 52) | Absent  (N = 169) |
| Recurrence in pStage II-III cases |  |  |  |
| Absent | 138 (62%) | 31 (60%) | 107 (63%) |
| Present | 83 (38%) | 21 (40%) | 62 (37%) |
|  |  |  |  |
| The first site of recurrence (R0-1) |  |  |  |
| Liver | 22 (27%) | 6 (29%) | 16 (26%) |
| Peritoneum | 18 (22%) | 1 ( 5%) | 17 (27%) |
| Lung | 12 (15%) | 4 (19%) | 8 (13%) |
| Bone | 3 ( 4%) | 0 | 3 ( 5%) |
| Adrenal | 2 ( 2%) | 0 | 2 ( 3%) |
| Brain | 1 ( 1%) | 0 | 1 ( 2%) |
| Anastomosis | 7 ( 8%) | 0 | 7 (11%) |
| Pleural | 2 ( 2%) | 1 ( 5%) | 1 ( 2%) |
| Other | 1 ( 1%) | 0 | 1 ( 2%) |
| Lymph node (LN) | 31 (37%) | 10 (48%) | 21 (34%) |
| Cervical | 4 (5%) | 1 | 3 |
| Mediastinal | 13 (16%) | 4 | 9 |
| Para-aortic | 14 (17%) | 3 | 11 |
| Abdominal | 6 ( 7%) | 4 | 2 |
| Unknown | 0 | 0 | 0 |

(%) indicates the proportion of cases among each group of the presence or absence of Barrett’s esophagus.

**SUPPLEMENTARY TABLE 4 Mortality of the patient with Barrett’s esophagus** **in all cases (N = 395)**

| Clinicopathological factors | Total No. |  | EGJ-cancer-specific survival | | |  | Relapse-free survival | | |  | Overall survival | | |
| --- | --- | --- | --- | --- | --- | --- | --- | --- | --- | --- | --- | --- | --- |
|  |  |  | No. of events | Univariate  HR  (95% CI) | Multivariate  HR  (95% CI) |  | No. of events | Univariate  HR  (95% CI) | Multivariate  HR  (95% CI) |  | No. of events | Univariate  HR  (95% CI) | Multivariate  HR  (95% CI) |
| Barrett’s esophagus |  |  |  |  |  |  |  |  |  |  |  |  |  |
| Present (vs. Absent) | 124 |  | 29 | 0.50 (0.32-0.74) | 1.16 (0.73-1.77) |  | 45 | 0.53 (0.38-0.73) | 0.96 (0.67-1.36) |  | 42 | 0.51 (0.36-0.71) | 0.95 (0.65-1.36) |
|  |  |  |  | ***P* = 0.0004** | *P* = 0.52 |  |  | ***P* <0.0001** | *P* = 0.83 |  |  | ***P* <0.0001** | *P* = 0.80 |

The multivariate, Cox proportional hazard regression model initially included gender, age, year of surgery, body mass index, tumor diameter, existence of Barrett’s esophagus, disease stage, tumor differentiation, lymphatic invasion, venous invasion, surgical approach, operative time, blood loss volume, blood transfusion, resection margin, preoperative complication, adjuvant chemotherapy. A backward elimination with a threshold of *P*=0.20 was used to select variables in the final model. CI, confidence interval; HR, hazard ratio.

**SUPPLEMENTARY TABLE 5 Mortality of the patient with Barrett’s esophagus in pStage II and III cases (N = 221)**

|  | Total No. |  | EGJ-cancer-specific survival | | |  | Relapse-free survival | | |  | Overall survival | | |
| --- | --- | --- | --- | --- | --- | --- | --- | --- | --- | --- | --- | --- | --- |
|  |  |  | No. of events | Univariate  HR  (95% CI) | Multivariate  HR  (95% CI) |  | No. of events | Univariate  HR  (95% CI) | Multivariate  HR  (95% CI) |  | No. of events | Univariate  HR  (95% CI) | Multivariate  HR  (95% CI) |
| Barrett’s esophagus |  |  |  |  |  |  |  |  |  |  |  |  |  |
| Present (vs. Absent) | 52 |  | 18 | 0.93 (0.53-1.55) | 1.05 (0.59-1.76) |  | 26 | 0.83 (0.53-1.26) | 0.92 (0.58-1.41) |  | 24 | 0.78 (0.48-1.20) | 0.88 (0.54-1.37) |
|  |  |  |  | *P* = 0.79 | *P* = 0.87 |  |  | *P* = 0.40 | *P* = 0.71 |  |  | *P* = 0.26 | *P* = 0.58 |

The multivariate Cox regression model included the same set of covariates selected as in **SUPPLEMENTARY TABLE 3**.

CI, confidence interval; HR, hazard ratio.
